# A 6-year retrospective study of clinical features, microbiology, and genomics of Shiga toxin-producing *Escherichia coli* in children who presented to a tertiary referral hospital in Costa Rica

**DOI:** 10.1128/spectrum.03056-23

**Published:** 2024-02-09

**Authors:** Cristian Perez-Corrales, Valeria Peralta-Barquero, Francisco Duarte-Martinez, Gletty Oropeza-Barrios

**Affiliations:** 1 División de Microbiología, Hospital Nacional de Niños “Dr. Carlos Saenz Herrera”, Caja Costarricense de Seguro Social, San José, Costa Rica; 2 Laboratorio Nacional de Referencia en Inocuidad Microbiológica de Alimentos, Instituto Costarricense de Investigación y Enseñanza en Nutrición y Salud, San José, Costa Rica; 3 Laboratorio Nacional de Referencia en Bacteriología, Instituto Costarricense de Investigación y Enseñanza en Nutrición y Salud, San José, Costa Rica; London Health Sciences Centre, London, Canada

**Keywords:** STEC, Shiga-toxin *Escherichia coli*, STEC genomic surveillance

## Abstract

Shiga-toxin-producing *Escherichia coli* (STEC) is associated with diarrhea and hemolytic uremic syndrome (HUS). STEC infections in Costa Rica are rarely reported in children. We gathered all the records of STEC infections in children documented at the National Children’s Hospital, a tertiary referral hospital, from 2015 to 2020. Clinical, microbiological, and genomic information were analyzed and summarized. A total of 3,768 diarrheal episodes were reviewed. Among them, 31 STEC were characterized (29 fecal, 1 urine, and 1 bloodstream infection). The prevalence of diarrheal disease due to STEC was estimated at 0.8% (*n* = 29/3,768), and HUS development was 6.4% (*n* = 2/31). The *stx1* gene was found in 77% (*n* = 24/31) of STEC strains. *In silico* genomic predictions revealed a predominant prevalence of serotype O118/O152:H2, accompanied by a cluster exhibiting allele differences ranging from 33 to 8, using a core-genome multilocus sequence typing (cgMLST) approach. This is the first study using a genomic approach for STEC infections in Costa Rica.

**IMPORTANCE:**

This study provides a comprehensive description of clinical, microbiological, genomic, and demographic data from patients who attended the only pediatric hospital in Costa Rica with Shiga-toxin-producing *Escherichia coli* (STEC) infections. Despite the low prevalence of STEC infections, we found a predominant serotype O118/O152:H2, highlighting the pivotal role of genomics in understanding the epidemiology of public health threats such as STEC. Employing a genomic approach for this pathogen for the first time in Costa Rica, we identified a higher prevalence of STEC in children under 2 years old, especially those with gastrointestinal comorbidities, residing in densely populated regions. Limitations such as potential geographic bias and lack of strains due to direct molecular diagnostics are acknowledged, emphasizing the need for continued surveillance to uncover the true extent of circulating serotypes and potential outbreaks in Costa Rica.

## INTRODUCTION

Each year, diarrheal disease causes death to more than half a million children under 5 years old (1, 2). Since 2013, the WHO and UNICEF Integrated Global Action Plan for the Prevention and Control of Pneumonia and Diarrhea proposed an integrated approach to reduce mortality from diarrhea in children less than 5 years old to fewer than one per 1,000 live births by 2025 (3). Diarrheagenic *Escherichia coli* is a major contributor to morbidity and mortality worldwide (4). Among them, Shiga-toxin-producing *Escherichia coli* (STEC) encompasses a diverse pathotype that can cause mild to bloody diarrhea and hemolytic uremic syndrome (HUS), characterized by hemolytic anemia and nephropathy, which is triggered by the action of Shiga toxins when absorbed into the systemic circulation and which can be exacerbated using antibiotics (4, 5). It is estimated that up to 10% of patients with STEC infection may develop HUS, with a case-fatality rate ranging from 3% to 5% (6). Cattle have been associated as a major reservoir of STEC affecting humans (7). STEC is transmitted to humans primarily through the consumption of contaminated foods, such as raw or undercooked ground meat products, raw milk, and contaminated raw vegetables and sprouts (1).

Classic bacterial pathogen detection methods such as culture, microscopy, and biochemical tests are laborious and time-consuming (4, 8). These assays are not useful for detecting STEC, since phenotypic assays based on virulence properties or molecular methods are required for the specific identification of virulence factors such as the Shiga-toxin genes *stx1* and *stx2* (9).

The continuous expansion in massive parallel sequencing (whole-genome sequencing, WGS) marks a groundbreaking leap forward by enhancing the capabilities to explore in more detail bacterial characteristics, including sequence type, serotype, genes linked to antimicrobial resistance, and phylogenetic relationships. It can also potentiate clinical practice and public health interventions, e.g., when archived data or bacterial isolates are used to determine phenotypic traits such as resistance mechanisms to antimicrobials, virulence factors, and association with outbreaks and mortality (10).

The National Children’s Hospital of Costa Rica (HNN-CCSS) is a tertiary referral hospital within the socialized medical care system and the only pediatric hospital in the country. STEC strains isolated by the National Laboratories require confirmation by the National Reference Center (NRC) for Bacteriology or the NRC for Microbiological Food Safety (Instituto Costarricense de Investigación y Enseñanza en Nutrición y Salud, Inciensa). Between 2005 and 2016, 52 cases of STEC infections were documented in children in Costa Rica, all from HNN-CCSS (11, 12).

Here, we provide the first genomic study of STEC in Costa Rica, based on a nationwide surveillance program using whole-genome sequencing. We also analyze the clinical profiles of the children from whom these STEC were recovered and describe possible associations between genomic findings, epidemiology, and illness.

## MATERIALS AND METHODS

### Demographic, clinical, microbiological, molecular, and epidemiological data

All data from the microbiological analysis of stool cultures and STEC identification between January 2015 and July 2020 in patients under 13 years of age were extracted from HNN-CCSS laboratory information systems: Labcore (Rochem Biocare, Bogotá, Colombia) and Copernico (Biomérieux, Marcy L’étoile, France) and collated with Microsoft Excel 365. The study included patients ranging from 0 to 13 years of age, reflecting the age range covered by the HNN-CCSS. All patients with STEC detection in at least one fecal or extraintestinal sample were selected. Health records were reviewed for each patient using a standardized form. Demographic (age, sex, and location/province), clinical (underlying diseases, hospitalization, peripheral blood tests, HUS development, and outcome), microbiologic (sample type and characteristics and susceptibility test), molecular and genomic (PCR results and whole-genome sequencing data) variables were analyzed.

### Laboratory workup

#### Bacterial culture and polymerase chain reaction

Pathogen screening was performed by culturing the stools in several media including *Campylobacter* agar, *Salmonella-Shigella* agar, blood agar supplemented with ampicillin (5%), and Tergitol-7 agar. The predominant growth of yellow lactose-fermenting colonies on Tergitol-7 agar (Thermo Scientific Oxoid) was selected for end-point PCR. DNA extraction was performed using Maxwell (Promega Corp, Madison, WI, USA) Cell-DNA extraction protocol according to the manufacturer’s instructions. PCR assays targeting STEC genes *eae*A, *stx*1, *stx*2 (STEC), and *rfbE* (O157) were conducted using primers and conditions previously described (11, 13). When available, samples with STEC detection through Filmarray GI Panel (BioFire Diagnostics, LLC, Salt Lake City, UT, USA) were also cultured in Tergitol-7 agar and were analyzed with the PCR assay mentioned before.

STEC from other sources were identified by PCR upon medical request due to clinical manifestations of the patients (unpaired kidney function) together with the isolation of *Escherichia coli*. Identified STEC isolates were derived for further confirmation analyses to the Inciensa. These analyses included verification of the genes by multiplex endpoint-PCR targeting *eae*A, *stx*1, *stx*2, and *rfb*O157 genes, as described by Leotta et al. (14)

#### Identification and antimicrobial susceptibility testing

Identification and antimicrobial susceptibility tests (AST) for PCR-confirmed STEC were performed by automated means using Vitek 2 GN (Gram-negative) and AST-577 cards, according to the manufacturer’s instructions (Biomérieux, Marcy L’étoile, France) and Clinical Laboratory Standards Institute breakpoints (15). The AST-577 card included the following antimicrobials: amikacin (AK), gentamicin (GN), ampicillin (AMP), ampicillin-sulbactam (AMS), cefalotin (CTN), cefotaxime (CTX), cephepime (FEP), imipenem (IP), meropenem (MP), ciprofloxacin (CIP), nitrofuraintoin (NIT), trimethoprim-sulfamethoxazole (TMP-STX), and piperacillin-tazobactam (P/T).

### Whole-genome sequencing

Whole-genome sequencing was performed in both facilities (HNN-CCSS and Inciensa) using the DNA extracted using the same protocol described for PCR assays. Illumina Nextera Flex (Illumina Inc., San Diego, CA, USA) kit and Illumina Miseq platforms were used for library preparation. At the HNN-CCSS, a 300 bp paired-end fragment protocol recommended by Illumina (Illumina Inc., San Diego, CA, USA) was used. The PulseNet International SOP (https://pulsenetinternational.org/protocols/wgs/) was executed at Inciensa for bioinformatic analyses. First, 250- or 300-bp sequence-read quality was checked with FastQC (16). Bioinformatic analyses were performed using BioNumerics v7.6.3 software (Applied Maths Inc., Biomérieux, Austin, TX, USA). Genome assembly was performed with the Applied Maths cloud-based calculation engine, and the resulting genomes were uploaded to NCBI (see the supplemental material). A wgMLST scheme for *Escherichia coli* was used to determine the allelic profile (i.e., unique sequences and their variation due to mutations produced by evolutionary events). Two algorithms were used for the identification of alleles: the assembly-free strategy found alleles, based on the raw sequence reads using a *k*-mer-based approach, and the assembly-based algorithm identified alleles based on the *de novo* SPAdes (17) assembled genomes using BLAST (18). Only alleles identified by both calling algorithms were considered present in the genome. *E. coli* functional genotyping plugin v1.2 was used for predicting phenotypic traits such as virulence factors, antimicrobial resistance, O/H predictions, prophages, and plasmid detection. For the cluster identification approach, a dendrogram was constructed using the categorical values similarity coefficient and the unweighted pair group method with arithmetic mean for hierarchical clustering.

### Statistical analyses

All data were recovered and collated using Microsoft Excel. Descriptive statistics were performed using Microsoft Excel. Statistics Kingdom online software was used for statistical analysis. Continuous variables were expressed as median and H-spread (interquartile range, IQR).

## RESULTS

**Fig 1:**
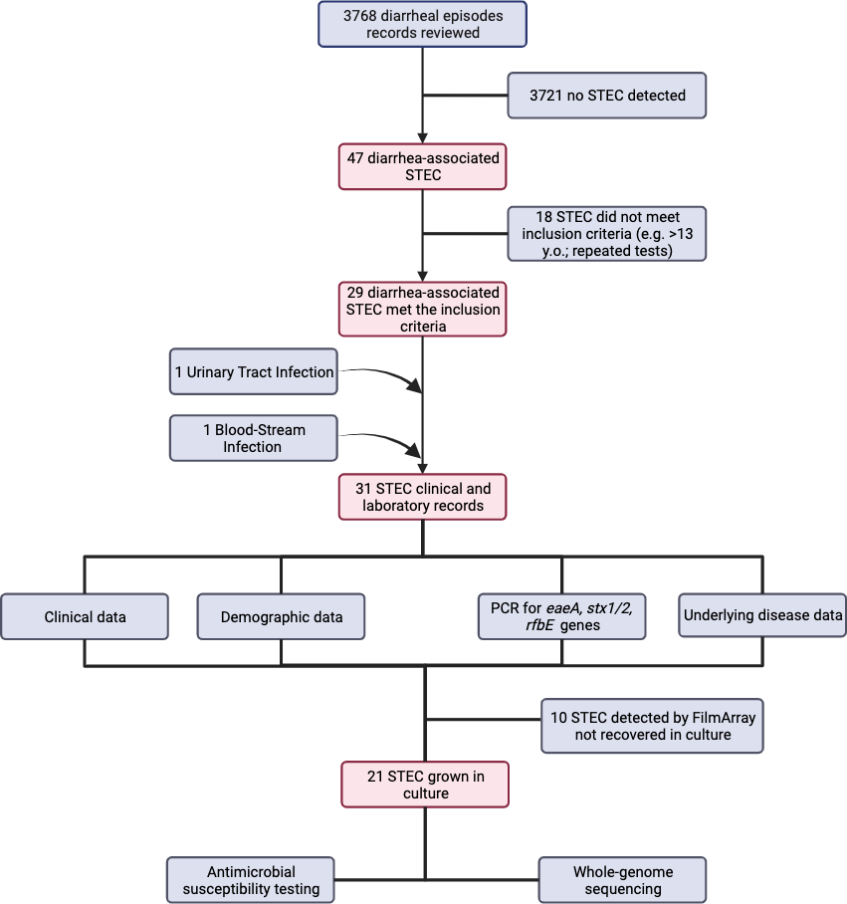
Flowchart showing the cases and the information available for the different variables included in the study. Image was created with BioRender.com.

A total of 3,768 records of stool culture were retrieved from the hospital databases consulted between January 2015 and July 2020. Among them, 49 (1.3%) STEC reports were documented [2015; *n* = 8 (0.2%)], [2016; *n* = 12 (0.3%)], [2017; *n* = 11 (0.3%)], [2018; *n* = 5 (0.1%)], [2019; *n* = 9 (0.2%)], and [2020; *n* = 4 (0.1%)]. After removing records according to the eligibility criteria, 31 cases were selected [29 fecal (0.8%), 1 blood, and 1 urine culture]. Additionally, 10 cases were diagnosed using BioFire Filmarray technologies, and the STEC strains were not recovered. The remaining 21 strains were derived for genomic sequencing. Frequencies were calculated according to the available data for each variable (see Fig. 1).

### Demographic results

The median age of the cases was 24 months (range 4–156; IQR: 66–11), distributed in 55% (*n* = 17) females and 45% (*n* = 14) males. Most cases (*n* = 22/31; 71%) were detected among patients who lived in the capital of the country, San José. The remaining cases were distributed mainly in the nearby provinces of Alajuela (*n* = 4; 13%) and Heredia (*n* = 3; 10%). One case (*n* = 1; 3%) was documented in Puntarenas and another in Guanacaste, outside the central valley, which congregates about 60% of the country’s population.

### Clinical results

Underlying diseases were identified in 65% (*n* = 20/31) and were mainly gastrointestinal (40%; *n* = 8) but also due to congenital malformations (20%; *n* = 4), onco-hematological (15%; *n* = 3), and kidney diseases (15%; *n* = 3). Hospitalization was required in 23% (*n* = 7/31) of the cases. Peripheral leukocyte count was available for 68% (*n* = 21/31), with a median value of 10.8 × 10e^3^ cells/mm^3^ (IQR 15–6.8), and 48% (*n* = 10/21) of leukocytes. The median hemoglobin was 12.2 g/dL (IQR 13.6–10.6). Two patients developed HUS (6.4%), and one of them died from complications of the disease. Another death was documented due to hypovolemic shock induced by severe diarrhea in a patient with concomitant detection of STEC, enteroaggregative *Escherichia coli*, and norovirus.

Diarrhea was the main manifestation where STEC was associated as a causative agent (94%; *n* = 29). Nonetheless, one case was determined by direct STEC isolation from blood and another from urine. Blood and mucus in diarrhea were documented in 79% (*n* = 23/29) of diarrheal cases.

### Identification and susceptibility to antimicrobials

Information on 21 of 31 STEC strains was available (Fig. 1). Susceptibility was 100% to AK, IP, MP, and NIT; 95% (*n* = 1/21) to P/T, CIP, and GN; 90% (*n* = 2/21) to CTX, CAZ, and FEP; 80% (*n* = 4/21) to CTN and TMP/STX; 67% (*n* = 7/21) to AMS and 62% (*n* = 8/21) to AMP. Additionally, 10% (*n* = 2/21) of strains expressed extended-spectrum beta-lactamase (ESBL), determined by the inhibition of resistance in the presence of clavulanate.

### Detection of *eae*, *stx*1 *stx*2, and *rfb*E genes

Stx1-encoding gene was found in 24/31 (77%) of the cases, while 7/31 (23%) were positive for *stx2*. Only one case (3%) was found to harbor both *stx1* and *stx2* genes. Intimin-encoding gene *eae* was detected in 15/31 (48%). There was no detection of the *rfb*E gene in the 31 patients included in the study.

### Whole-genome sequencing

#### *In silico* predictions

**Fig 2:**
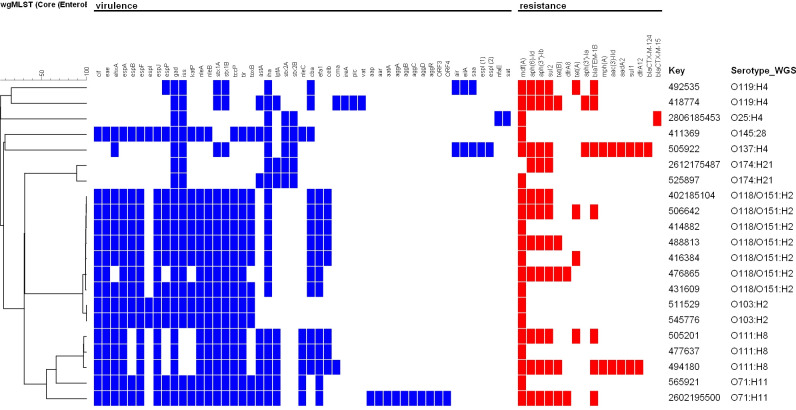
Dendogram of 21 STEC strains included in the study, showing effector proteins, virulence factors, toxins, and serotypes found from whole-genome sequencing analyses.

The most commonly predicted serotype was O118/O152:H2 (*n* = 7/21, 33%) followed by O111:H8 (*n* = 3/21, 14%) and two for the following: O71:H11, O103:H2, O174:H21, and O119:H4. The remaining three strains had predicted serotypes O145:H28, O25:H4, and O137:H4, respectively. ESBL-conferring genes were identified as CTX-M15 for the strain serotype O25:H4 and CTX-M124 for the strain O137:H4 (Fig. 2). These two strains were successfully detected as ESBL positive by phenotypic methods as mentioned before. Similarly, *sul1, sul2,* and *dfr*A12 genes were found in those strains that showed phenotypical resistance to TMP/STX, and *bla*_TEM_ was detected among isolates resistant to ampicillin.

#### Genomic similarity analysis and serotyping

**Fig 3:**
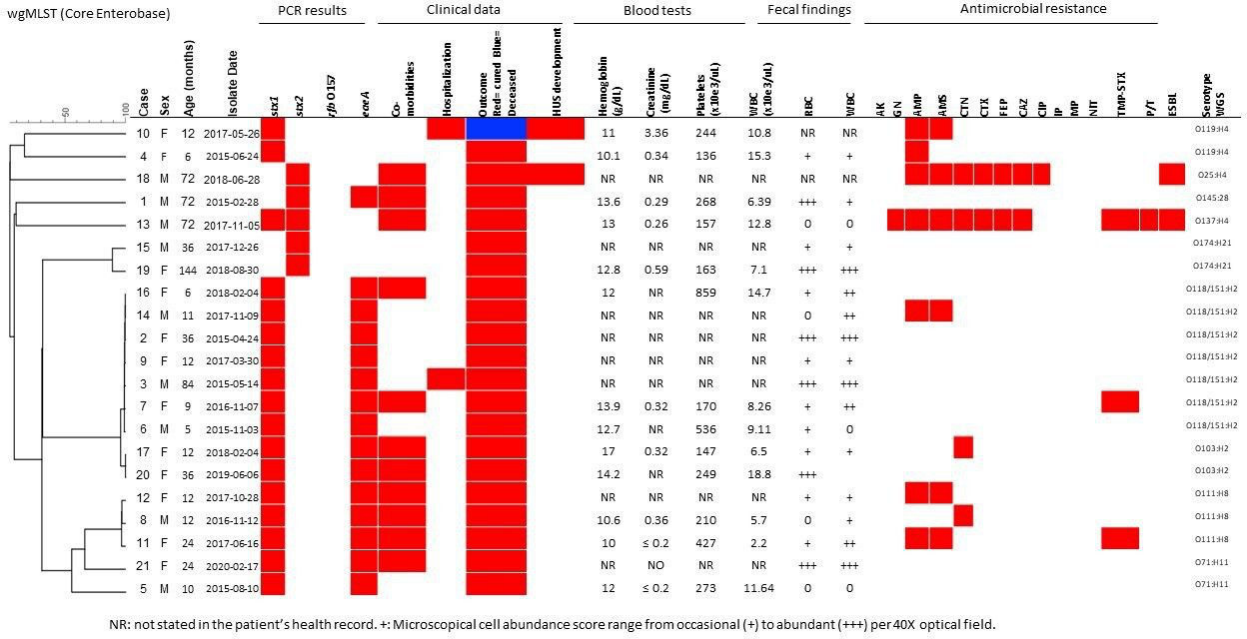
Dendogram of the 21 STEC strains included in the study, showing demographic, clinical, and antimicrobial resistance data.

Two clusters were identified according to the core genome analysis. A main cluster was constituted by seven strains that span over 4 years (from 2015 to 2018) and belong to the O118/O152:H2 serotype, exhibiting a maximum difference of 33 alleles. Within this cluster, three strains showed less than eight alleles of difference. All O118/O152:H2 strains harbored in common the *mdf*A, *stx1,* and *eaeA* genes encoding a membrane protein belonging to the major facilitator superfamily of transport proteins, Shiga-like toxin, and intimin protein, respectively. A predominance in children under 1 year of age (5/7, 71%) was observed, although non-statistical significance was established (Chi square 1.52, *P* = 0.21). The two remaining cases were detected in 3- and 7-year-old children. A second cluster with two *E. coli* O103:H2 strains was also identified, differing from each other by a single allele in the core genome analyses, even when recovered over a 1-year difference (Fig. 3). All clustered isolates were recovered from children living in different areas of the central valley.

## DISCUSSION

In this study, we could establish for the first time a WGS-based study of STEC in Costa Rica. The genomic approach has been demonstrated to be a powerful tool to better investigate and understand the epidemiology of public health threats. Our findings provide further evidence of a modest circulation of STEC within the pediatric population of Costa Rica, mainly in children under 2 years of age who exhibited other gastrointestinal co-morbidities and within the most densely populated regions (11, 12). The main clinical presentation we found was bloody diarrhea with discrete leukocytosis. We could not recover 10 strains detected by molecular means, even along with traditional culture methods. This might bias the prevalence estimation, due to the exclusion of cases that did not meet the predefined inclusion criteria. Rapid molecular strategies provide significant help in establishing the etiology of gastroenteritis, but it may be difficult to recover the agent for downstream analyses such as genomic-based epidemiology and outbreak investigation. Using a combination of conventional PCR and a validated genomic approach (19), we inferred *in silico* serotypes, virulence factors, and similarities among the strains under study. We found a predominant and clustered circulation of the O118/O152:H2 serotype, and also a discrete cluster was configured by O103:H2 strains. These clusters did not exhibit spatial-temporal association, limiting the probability of being related to an outbreak. While no statistical association was demonstrated, five out of seven cases were detected with children under 1 year of age. This finding highlights the importance of continuing these studies, as infants can be affected in large proportions, as seen in the most recent outbreak in Alberta, Canada, with more than 400 cases described (20). Notably, we did not find cases of O157:H7 in this study, in contrast with a study among 77 pediatric patients conducted in Argentina that found O157:H7 and O145:NM as the most prevalent (21). Of note, a limitation of this study is the potential geographic bias inherent to the hospital location. The NCH-CCSS is situated in the capital, and as a result, our findings may be skewed toward cases originating from this central region. Children residing far from the capital, particularly those with STEC diarrheal disease characterized by milder symptoms, may be less likely to seek medical attention at this specific facility.

The low prevalence of STEC infections found could suggest that the incidence of these infections may be lower in Costa Rica than in other countries. However, the high rate of underlying diseases among STEC-infected patients and the occurrence of severe complications such as HUS and death highlight the importance of continued surveillance and prevention measures. Whole-genome sequencing proved to be a valuable tool for identifying the serotypes, genotypes, and phylogenetic relationships of STEC strains in Costa Rica. In conclusion, this study provides insights into the epidemiology, clinical characteristics, and genetic diversity of STEC in Costa Rica. More studies are necessary to establish whether our findings are consistent or represent just the tip of the iceberg. Strengthening of the nationwide surveillance strategy could unveil the presence of other circulating serotypes, clusters, and outbreaks that could not be accurately determined due to the limitations of this study.

## Data Availability

Illumina short reads from this study were deposited at the Sequence Read Archive (SRA) at the NCBI and are available under the BioSample accession numbers SAMN38391730, SAMN38391731, SAMN38391732, SAMN38391733, SAMN38391734, SAMN38391789, SAMN38391790, SAMN38391791, SAMN38391792, SAMN38391793, SAMN38391909, SAMN38391910, SAMN38391911, SAMN38391912, SAMN38391913, SAMN38391914, SAMN38391915, SAMN38391916, SAMN38391917, SAMN38391918, and SAMN38391919, under the BioProject accession number PRJNA962092.

## SUPPLEMENTAL MATERIAL

Table S1 spectrum.03056-23-s0001.xlsx Accession numbers and sequencing metrics for studied strains.

ASM does not own the copyrights to Supplemental Material that may be linked to, or accessed through, an article. The authors have granted ASM a non-exclusive, world-wide license to publish the Supplemental Material files. Please contact the corresponding author directly for reuse.
